# Examining the inorganic elemental composition of lobster phyllosoma (*Panulirus ornatus*) with X-ray fluorescence microscopy

**DOI:** 10.1093/mtomcs/mfad038

**Published:** 2023-06-16

**Authors:** Daniel R McDougall, Robert Deas, Daryl L Howard, Quinn P Fitzgibbon, Gregory G Smith, Andrew G Jeffs, Duncan J McGillivray

**Affiliations:** School of Chemical Sciences, University of Auckland, Private Bag 92019, Auckland, New Zealand; School of Chemical Sciences, University of Auckland, Private Bag 92019, Auckland, New Zealand; Australian Synchrotron, 800 Blackburn Road, Clayton, Victoria 3168, Australia; Institute for Marine and Antarctic Studies (IMAS), University of Tasmania, Private Bay 49, Hobart, Tasmania, Australia; Institute for Marine and Antarctic Studies (IMAS), University of Tasmania, Private Bay 49, Hobart, Tasmania, Australia; Institute of Marine Science, University of Auckland, Private Bay 92019, Auckland, New Zealand; School of Chemical Sciences, University of Auckland, Private Bag 92019, Auckland, New Zealand

**Keywords:** heavy metals, aquaculture, marine biology, phyllosoma, lobster, bromine, XFM

## Abstract

The ornate spiny rock lobster, *Panulirus ornatus*, is an attractive candidate for aquaculture. The larval stages of spiny lobsters, known as phyllosoma, are complex with many developmental stages. Very little is known about the inorganic element composition of phyllosoma. In this study, a novel method using synchrotron X-ray fluorescence microscopy (XFM) was applied to investigate the distributions of metals potassium (K), calcium (Ca), copper (Cu), zinc (Zn), the metalloid arsenic (As), and nonmetal bromine (Br) within individual phyllosoma at stages 3, 4, and 8 of their development. For the first time, 1 µm resolution synchrotron XFM images of whole phyllosoma as well as closer examinations of their eyes, mouths, setae, and tails were obtained. Elements accumulated in certain locations within phyllosoma, providing insight into their likely biological role for these organisms. This information may be useful for the application of dietary supplementation in the future to closed larval cycle lobster aquaculture operations.

## Graphical Abstract

**Graphical Abstract:**
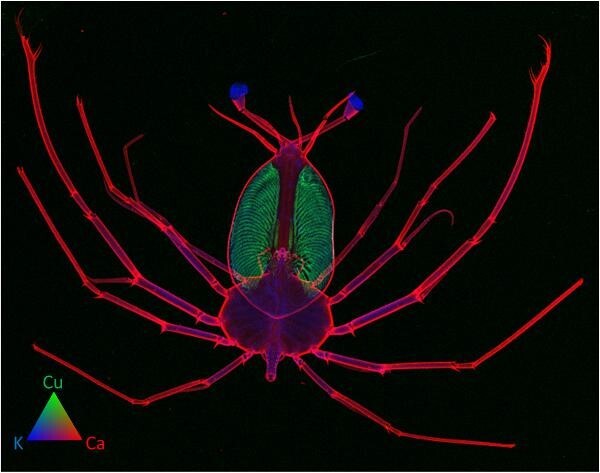
Distribution of potassium (K, blue), copper (Cu, green) and calcium (Ca, red) within a stage 8 *P. ornatus* lobster phyllosoma.

## Introduction

The ornate spiny rock lobster, *Panulirus ornatus*, is one of the most highly prized and valuable seafood species in the world. The wild population of this species extends throughout the tropical Indo-Pacific region from the eastern coast of Africa through to the western Pacific Ocean, including southern parts of Japan to northeastern Australia.^1^ However, it is evident that globally the fishery for wild spiny lobsters is fully exploited with landing yields plateauing in recent decades despite improved technology and increased fishing effort.^2^ The ornate spiny lobster is an attractive candidate for aquaculture due to many favorable attributes including high value, rapid growth, high fecundity, and communal behaviour.^3^ Aquaculture of the ornate lobster has been established in many parts of Southeast Asia, which are reliant on the harvesting of the post-larval settlement stage and early juveniles to provide the seed for ongrowing.^4,5^ The widespread harvesting of millions of early ornate lobsters to support this aquaculture activity is creating concerns over ecological sustainability.^6^ In addition, the harvested wild early stages of lobsters are known to vary considerably in both quality and quantity, leading to uncertainty and unreliability for subsequent aquaculture production.^7,8^ The establishment of hatchery technology that enables the rearing of juvenile ornate lobsters from eggs through their multiple larval stages is expected to help avoid overexploitation of wild stocks and enable sustainable aquaculture production.^9^ There are good future prospects for the aquaculture production of ornate lobsters to meet the increasing global demand for lobster products despite a constrained existing supply from wild fisheries.^10^ However, considerable further research and understanding of the culture requirements are needed.

**Fig. 1:**
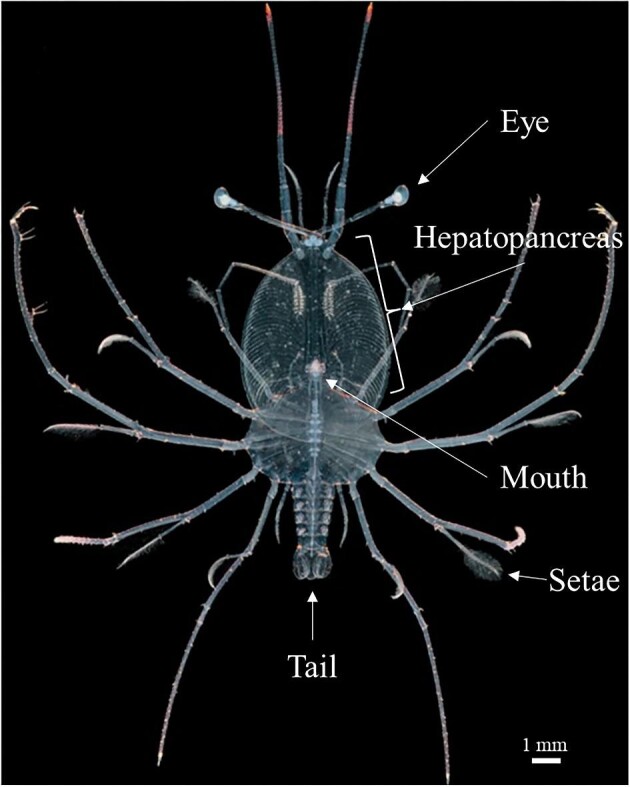
Labelled light microscope image of a *P. ornatus* phyllosomata. Adapted from image obtained with permission from ARC Research Hub for Sustainable Onshore Lobster Aquaculture.

The larval stage of spiny lobsters are known as phyllosoma (phyllosomata individual) (Latin etymology: leaf-like body) due to their characteristic dorso–ventrally flattened morphology (Fig. 1).

The life cycle of the ornate lobster is complex consisting of 11 morphologically distinct planktonic phyllosoma stages, followed by a transitional planktonic post-larval puerulus stage, a benthic post-puerulus, a juvenile and then an adult stage.^11^ In the wild the phyllosoma undergo most of their development in offshore oceanic waters, which is thought to be the result of an evolutionary strategy to avoid the high abundance of planktonic predators in coastal waters.^12^

Very little is known about the mineral requirements of phyllosoma. Much of their development occurs in the vast open ocean, which makes sampling of wild populations difficult, time consuming, and costly. The metal compositions of aquaculture reared spiny lobsters have not been studied to date, with the presumption that requirements are satisfied by the culture water and feed without the need for dietary supplementation.^13^ Potassium, calcium, copper, and zinc are all required by crustaceans for use in tissue construction and essential metabolic functions; however, it is unclear to what extent they need to be supplemented in aquaculture feeds.^14,15^

Potassium is key for the ionic conductances in neurons, e.g. the transient potassium current is particularly important in regulating the cyclic frequency of motor patterns in the pyloric circuit of the stomatogastric ganglion in lobsters.^16^ Kanazawa *et al.*^17^ reported that diets containing 0.9% potassium improved growth of prawns compared with diets containing 1.8% potassium. Calcium has an important structural role incorporated in the chitin-based exoskeleton of crustaceans as calcium carbonate, as well as its involvement in several metabolic processes.^15^ It is assumed that seawater always contains sufficient amounts of potassium and calcium to satisfy the physiological needs of aquatic organisms, making the necessity of dietary supplementation unlikely.^15,18^

Copper is essential as a cofactor with respiratory proteins called hemocyanins, which are the oxygen carrying pigments in the hemolymph of a wide variety of invertebrates, including lobsters.^19^ Copper also functions in combination with several metabolically important enzymes and processes.^20^ Several studies have shown that crustaceans cannot meet their physiological copper requirements from seawater alone and that a dietary source is required for optimal growth and development.^15,17,21^ However, among aquatic invertebrates, crustaceans are more susceptible to copper toxicity, and sub-lethal levels of copper have been reported to induce morphological, physiological, and chromosomal changes in Panulirus *homarus*.^22^

Zinc is required for normal growth, development, and function in all animal species.^23^ It functions as a cofactor for a myriad of enzymes involved in key metabolic processes.^24^ However, zinc concentrations can become toxic for lobsters at sufficient levels. For example, seawater zinc concentrations of 18.5 µg L^−1^ have been associated with chronic eyestalk molt deformities in spiny lobster (*Sagmariasus verreauxi*) phyllosoma.^25^

Arsenic is typically found in higher concentrations in shrimp, crabs, and lobsters than in other aquatic organisms.^26^ For example, in a study of total arsenic in fish, molluscs, and crustaceans collected from Cienfugos Bay in Cuba, mean concentrations were 10.2, 22, and 26.5 µg g^−1^ (dry weight), respectively.^26^ Arsenic is infamous for its harmful toxic effects, which are strongly dependent on what the arsenic is associated with.^27–29^ For example, arsenic bearing minerals commonly found in nature such as the arsenic sulfides: relagar (AsS), orpiment (As_2_S_3_), arsenopyrite [Fe.(AsS)], nicolite (NiAs), cobaltite (CoAsS), enargite (CuAsS_4_), and tennantite [(Cu, Fe)_12_As_4_S_13_] have limited solubility, and therefore a low toxicity. Nevertheless, oxidation of these insoluble forms can result in the formation of soluble and highly toxic arsenites and arsenates (where arsenic has +3 and +5 oxidation states, respectively).^28^ However, arsenobetaine is the predominant arsenic compound found in marine animals, which has been shown to have a relatively low toxicity to aquatic organisms.^30^

Bromine is an essential element for animals, particularly through its role in the crosslinking of collagen.^31^ Bromine has also been shown to be an important component of the noncalcified chitin-based exoskeleton of crustaceans.^32^ Furthermore, Khan *et al.*^33^ found that halogens, such as bromine, are scarce at the tip of the jaws of the *Nereis* polychaetes, where wear resistance properties are most needed, and present in high concentrations at the base. Bromine also has been observed to accumulate in the mouth parts and setae of crustaceans, and furthermore throughout the whole organism including the antenna, pleopods (paddling limbs), and uropods (tails).^34^ Bromine is the eighth most abundant element in the ocean after chlorine, sodium, magnesium, calcium, potassium, and carbon^35^, forming compounds that are highly soluble in seawater and as a result is typically present in higher concentrations in marine life. Marine organisms are the main source of around 1600 different organobromine compounds, which are produced by several species of marine algae and animals.^36^ These organobromine compounds vary in toxicity at sufficient concentrations depending on the species of crustacean, with average toxicities roughly around 8.8, 68, and 137 mg L^−1^ for Copepoda, Cladocera, and Anostraca, respectively.^37^

The hepatopancreas of adult lobsters is internationally certified as a biological reference material and is commonly used for elemental speciation analyses (TORT-1, TORT-2). In a study comparing the metal content of adult *P. ornatus* muscle tissue when fed either fish (species not named) or green mussels (*Perna viridis*), the concentrations of copper, zinc, and arsenic were not significantly different, with concentrations ranging from 0.8 to 1.4, 9.6 to 24.4, and 0.17 to 0.48 µg g^−1^ (wet weight) for copper, zinc, and arsenic, respectively.^38^ However, it is well known that the trace metal requirements and toxicities of trace metals in particular for shellfish vary significantly depending on their stage of development, with the earlier stages considerably more vulnerable to trace metal toxicity in particular.^39,40^ Therefore, making comparisons between the requirements or toxicities of trace elements for adult and larval stages is not the best practice for developing a true understanding.

Studies of the elemental requirements of phyllosoma stage separately to the adult stage as they progress through the larval cycle are therefore crucial to determine which inorganic elements are essential, to understand their biological role, and quantify the concentrations required for healthy development. From this knowledge, dietary supplementation can be optimized or certain elements can be targeted in water treatment for closed larval cycle aquaculture if necessary.

This study capitalizes on the recent advances in the hatchery production of spiny lobsters. Access to cultured phyllosoma provides an opportunity to allow the first examination of inorganic element distribution during different stages of phyllosoma development, using synchrotron X-ray fluorescence microscopy.

## Methods

### Phyllosoma collection and developmental assessment

Larvae of *P. ornatus* were reared according to the proprietary rearing procedure at the Institute of Marine and Antarctic Studies (IMAS) aquaculture research facility in Taroona near the city of Hobart in Tasmania. This facility is situated on the western bank of the Derwent River embayment leading into the Tasman Sea. The seawater used for larval rearing was pumped ashore, mechanically filtered to 25 µm, treated with ozone and passed through activated carbon before use in onshore larval rearing tanks. All seawater was treated in exactly the same manner for all larval rearing tanks.

Ornate lobster larvae from two different tanks were collected and placed flat in a petri dish and observed under a light microscope to determine the stage of development through characteristic morphological features.^11,41^ One tank contained stage 3 and stage 4 larvae reared together, while the other contained stage 8 larvae. Three stage 3 larvae had reached instar 3 and the other three had reached instar 1 only. All seven stage 4 larvae had reached instar 2. The nine stage 8 larvae were varied in development, four had reached instar 2, four had reached instar 3, one had reached instar 4, and one had reached instar 5.

### Inductively coupled plasma–mass spectrometry

Seawater samples from the two different tanks were collected to assess their metal concentrations. All seawater samples taken for metal analysis were diluted 20 times with trace metal pure analytical grade 2% HNO_3_ prior to analysis. The samples were then analysed with inductively coupled plasma–mass spectrometry (ICP–MS) (7700x, Agilent, Santa Clara, CA, USA) and the concentrations of trace elements copper (Cu), zinc (Zn), and arsenic (As) were back-calculated for the original samples when present above detection limits. The ICP–MS was operated in helium mode to reduce polyatomic interferences. Calibration standards were prepared in a matrix-matched solution from 1000 ppm single element standards (Peak Performance, CPI International, Santa Rosa, CA, USA) as well as a blank matrix-matched solution containing no single element standards or seawater. An online internal standard of 20 ppb yttrium (Y) and terbium (Tb) was used to monitor and correct for instrument drift and matrix effects. Blanks with 2% HNO_3_ alone were analysed to observe the background metal content. The ICP–MS measured each sample 10 times and provides an average concentration detected in each replicate.

### X-ray fluorescence microscope sample preparation

A simple method was used for the preparation of samples ready for examination under an X-ray fluorescence microscope (XFM) given that previous research has found that minimizing the preparation steps of biological samples for analysis of elemental content is vital to ensure that the elements are in as close to their native state as possible.^42–44^ All samples were prepared in the same manner as follows: With tweezers, an individual phyllosomata was carefully placed flat on a 40 µm nylon mesh filter. The phyllosomata was then rinsed with deionized water using a Pasteur pipette to remove salt from their exterior. Then the phyllosomata was carefully transferred with tweezers and stuck flat on kapton adhesive tape, adhesive side up (3DEA-FISAG Ltd.). The adhesive immobilized the phyllosoma in this state. The tape is composed of a 1 mil (25.4 µm) thick polyimide film and a 1.5 mil (38.1 µm) silicone adhesive layer. All phyllosoma were then freeze-dried in this state overnight at 0.027 mBar and −53°C. The samples were carefully observed while the pressure was lowered and did not move or unstick from the Kapton tape as this happened. The larvae were also checked for ice crystal formation under an optical microscope as ice crystal formation could have caused tissue damage. Ice crystals were not observed, suggesting that the water within the samples was frozen in an amorphous state prior to sublimation under vacuum, leaving the original shape and structure of the samples intact.

### XFM sample examination

The monochromatic X-ray beam on the XFM was focused down to a spot size of approximately 2 × 2 μm using the Kirkpatrick-Baez-mirror system at the XFM beamline at the Australian Synchrotron.^45^ An incident photon energy of 18.5 keV on the XFM enabled the analysis of elements ranging from P to Zr via K-shell excitation and to U via L-shell excitation. For each measurement, phyllosomata were located on the Kapton tape and coordinates for each specimen were obtained and set for scanning. Each phyllosomata was scanned continuously through the focused beam and X-ray fluorescence spectra were collected using the Maia 384D22 detector.^46^ Then particular areas of interest were identified, coordinates ascertained, and higher resolution scans conducted. The highest resolution scans were recorded at 1 × 1 µm and 0.5 × 0.5 µm pixel sizes, effectively oversampling the focused beam spot size. The analysis of the raw fluorescence spectra was done using GeoPIXE software.^47^ Standards were scanned and fluorescence counts for the known concentrations in the standards were used in conjunction with the software fitting of fluorescence spectra to determine concentrations in the samples. Elements that were unmistakably present based on clear, distinct, characteristic X-ray fluorescence signals in the fluorescence spectra were potassium (K), calcium (Ca), copper (Cu), zinc (Zn), arsenic (As), and bromine (Br) in each phyllosomata examined. It is important to note that software fitting assumed a uniform sample thickness of 40 µm based on an average of measured thicknesses throughout the phyllosoma, and a sample density based on the theoretical density of a combination of cellulose, calcium carbonate and shell protein (see Supplementary Information for more detail). This provided semi-quantitative concentrations in units of microgram per gram of freeze-dried phyllosomata (µg g^−1^); however, there were certainly variations in thickness and composition depending on the locations investigated, which affected the accuracy of concentration quantification. Using GeoPIXE software, the area covered by an individual phyllosomata eye, mouth, hepatopancreas, and tail was selected and the mean concentrations of each of the six elements for those areas were recorded in units of micrograms per gram. The mean concentration (±S.E.) for each element for four different anatomical regions (eye, mouth, hepatopancreas, and tail) from all phyllosoma from each of the three stages of development (3, 4, and 8) was calculated. The possibility of artefacts resulting from X-ray-induced damage was assessed through inspection of large-area scans taken before and after fine detailed scanning. Negligible differences in count numbers between the before and after scans were interpreted to indicate that there was no significant elemental redistribution resulting from the measurement procedure.

### Statistical analyses

All phyllosoma elemental concentration data from XFM were tested for normality and homogeneity of variance prior to analysis and if compliant a one-way ANOVA was used to test for differences among the three developmental stages and among the four different anatomical regions. If the data were found to be noncompliant (i.e. failed either Shapiro–Wilk's or Bartlett's tests) they were log transformed and rechecked for compliance. If they remained noncompliant, a Kruskal–Wallis test was used to compare the results among treatments. Where the ANOVA or Kruskal–Wallis tests were significant, pairwise comparisons of means were conducted with Tukey's tests.

## Results

### X-ray fluorescence microscopy images

#### Stage 3

**Fig. 2:**
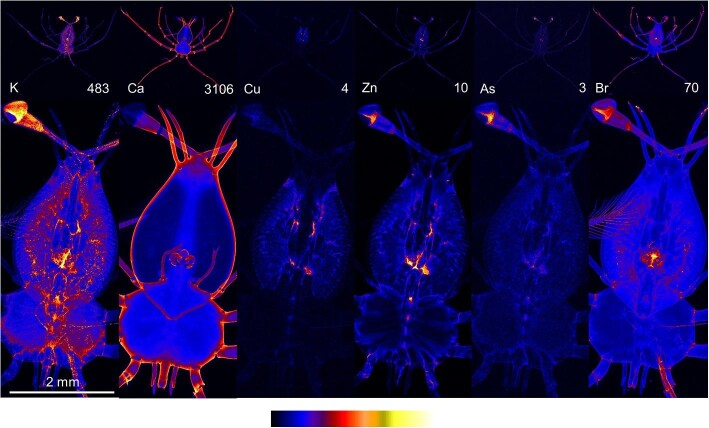
X-ray fluorescence microscopy images of a representative stage 3 phyllosomata. Whole organism (upper) and higher resolution scan (lower) images are shown for the distributions of potassium (K), calcium (Ca), copper (Cu), zinc (Zn), arsenic (As), and bromine (Br). The maximal elemental areal density (units of µg cm^−2^) is given in each image.

At stage 3 of development, phyllosoma had the highest concentrations of potassium in the eye and mouth (Fig. 2). The calcium distribution emphasized the calcium-based exoskeleton around the body, eye stalks, antennae, and legs. The highest copper concentrations were found in the hepatopancreas. Zinc had accumulated in the eyes and the mouth, and tended to localize in the joints of the appendages. The highest concentrations of arsenic were located in the eyes. Bromine concentrations were elevated in the eyes but the highest concentrations were located in the center of the mouth and on setae at the ends of the pereopods.

#### Stage 4

**Fig. 3:**
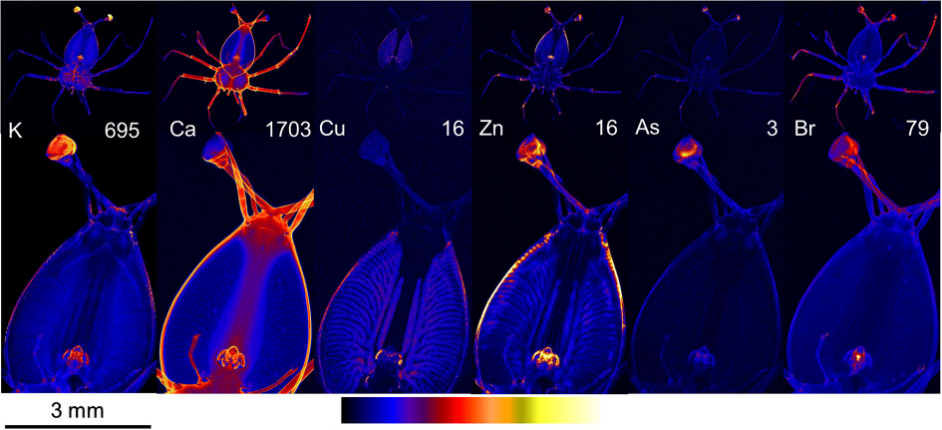
X-ray fluorescence microscopy images of a representative stage 4 phyllosomata. Whole organism (upper) and higher resolution scan (lower) images are shown for the distributions of potassium (K), calcium (Ca), copper (Cu), zinc (Zn), arsenic (As), and bromine (Br). The maximal elemental areal density (units of µg cm^−2^) is given in each image.

At stage 4 of development (Fig. 3), similar distributions of targeted elements were observed to those in stage 3, but there was an increase in contrast and signal intensity in high concentration areas. Potassium and arsenic accumulated in the eyes, calcium in the exoskeleton, copper in the hepatopancreas, and zinc in the eyes, mouth, and leg joints. The branched structure of the gut was clearest with copper distribution mapping. Bromine concentrations were raised in the eyes and in the center of the mouth.

#### Stage 8

**Fig. 4:**
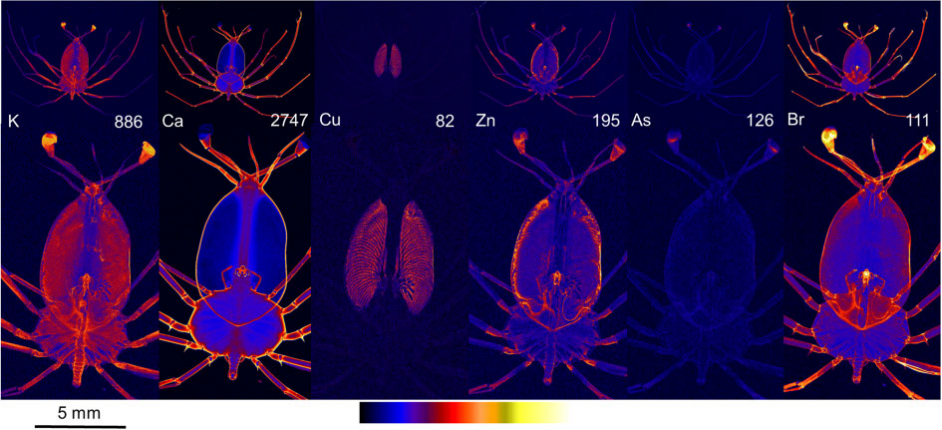
X-ray fluorescence microscopy images of a representative stage 8 phyllosomata. Whole organism (upper) and higher resolution scan (lower) images are shown for the distributions of potassium (K), calcium (Ca), copper (Cu), zinc (Zn), arsenic (As), and bromine (Br). The maximal elemental areal density (units of µg cm^−2^) is given in each image.

At stage 8 of development (Fig. 4), there was a further increase in contrast and signal intensity for high concentration areas compared to stages 3 and 4. In particular, the relative concentrations of copper in the hepatopancreas were much higher than the rest of the phyllosomata (Fig. 4, Cu).

#### Eye

**Fig. 5:**
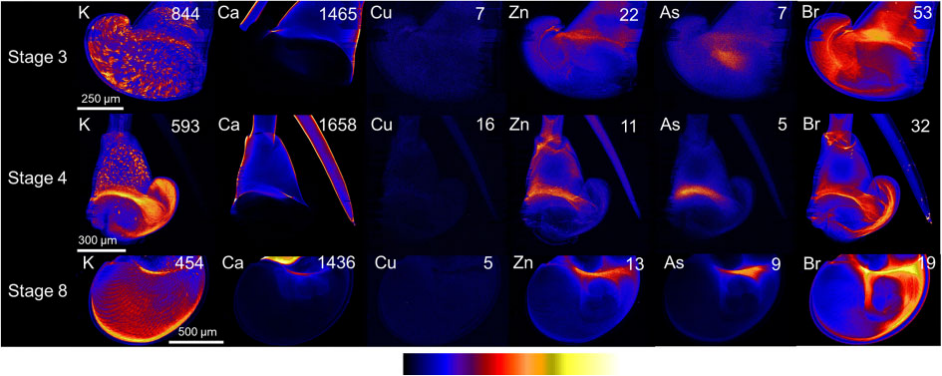
X-ray fluorescence microscopy images of representative stages 3, 4, and 8 phyllosomata eye. Whole organism (upper) and higher resolution scan (lower) images are shown for the distributions of potassium (K), calcium (Ca), copper (Cu), zinc (Zn), arsenic (As), and bromine (Br). The maximal elemental areal density (units of µg cm^−2^) is given in each image.

On closer inspection, the eyes had hotspots of potassium dotted throughout at stage 3 and stage 4 (Fig. 5). Potassium and zinc maps show the outlines of the tessellated compound eye at stage 8. There was a correlation among the distributions of zinc, arsenic, and bromine in the eyes at all three stages.

#### Mouth

**Fig. 6:**
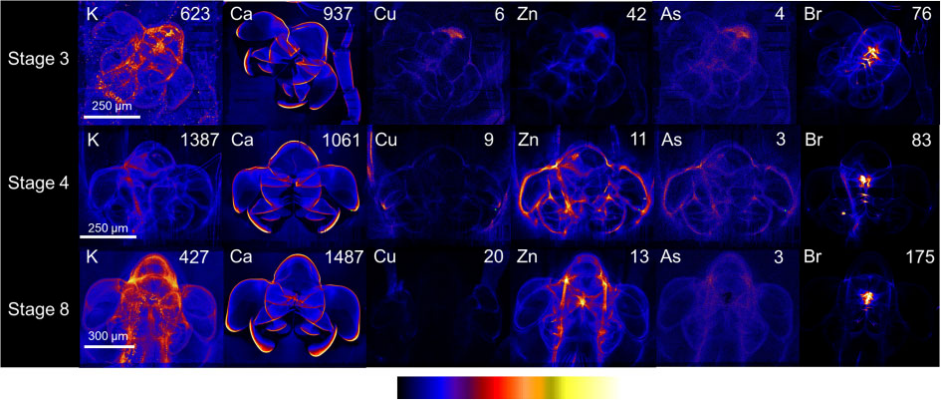
X-ray fluorescence microscopy images of representative stages 3, 4, and 8 phyllosomata mouth. Images are shown for the distributions of potassium (K), calcium (Ca), copper (Cu), zinc (Zn), arsenic (As), and bromine (Br). The maximal elemental areal density (units of µg cm^−2^) is given in each image.

At all stages, calcium distributions clearly outlined the mouth parts of phyllosoma and bromine concentrations were highest at the center of the mouth (Fig. 6). There was a increase in accumulation of zinc in specific areas of the mouth as the phyllosoma developed from stage 3 to stage 4 and stage 8.

#### Setae

**Fig. 7:**
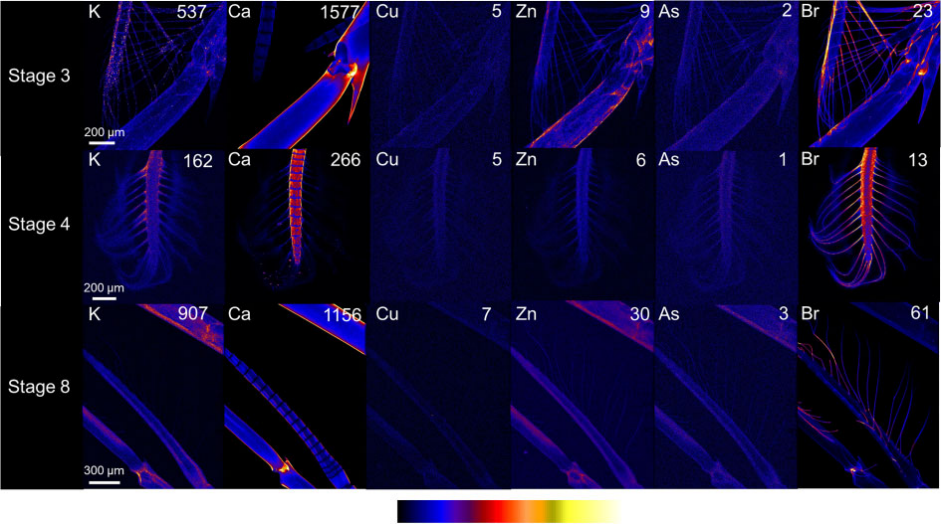
X-ray fluorescence microscopy images of representative stages 3, 4, and 8 phyllosomata setae. Images are shown for the distributions of potassium (K), calcium (Ca), copper (Cu), zinc (Zn), arsenic (As), and bromine (Br). The maximal elemental areal density (units of µg cm^−2^) is given in each image.

Closer examination of the appendages of phyllosoma at all stages revealed strong bromine accumulations within the hair-like setae extending from the appendages (Fig. 7). Potassium, copper, zinc, and arsenic distributions in the setae became less apparent as the phyllosoma developed from stage 3 and stage 4 to stage 8.

#### Tail

**Fig. 8:**
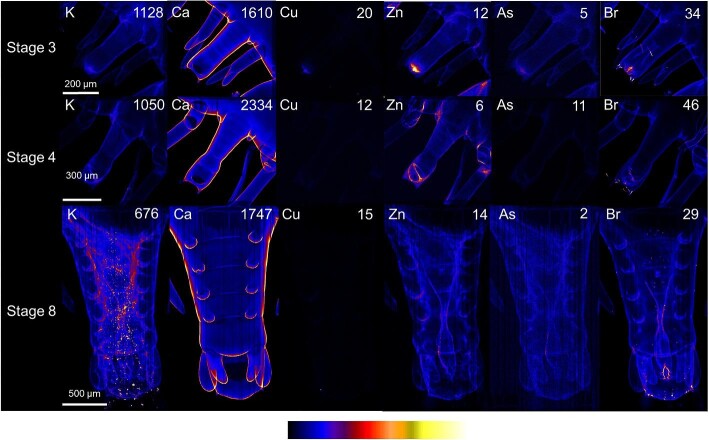
X-ray fluorescence microscopy images of representative stages 3, 4, and 8 phyllosomata tail. Images are shown for the distributions of potassium (K), calcium (Ca), copper (Cu), zinc (Zn), arsenic (As), and bromine (Br). The maximal elemental areal density (units of µg cm^−2^) is given in each image.

Calcium maps showed segmentation and uropods on the tail and their increasing complexity as the phyllosoma develop (Fig. 8). Zinc and bromine distributions showed the internal gut passage within the tail, with raised levels of these elements in the gut tissue, which was more developed at stage 8 compared to stages 3 and 4.

## Discussion

With the development of hatchery technology for the larval rearing of ornate lobsters from egg to juvenile, phyllosoma are now reared in controlled and optimized conditions for healthy and fast development, allowing studies on their inorganic element composition. These elements can either be sourced from the rearing water, or through the diet. This study is the first to map the distributions of elements within phyllosoma, providing much needed fundamental understanding about phyllosoma inorganic biochemistry.

Changes in concentration of elements with development or with changes in the area investigated do not confirm or deny a change in requirement for that element. For example, observation of higher concentrations of a certain element in a certain area does not necessarily mean that the element is required there. However, given the lack of current knowledge about the mineral requirements of phyllosoma, and the knowledge already available from observation of the mineral requirements of other species of aquatic invertebrates, these measurements may provide new insights into the potential uses of these elements by phyllosoma.

The elements with higher abundance in seawater such as potassium, calcium, and bromine, were all accumulated in certain areas of phyllosoma. The highest concentrations of potassium were located in the eye, where light receptors required for sensing are abundant. Potassium is key for cell signaling through neural networks via ionic conductance. Potassium is known to be important for motor function in the stomatogastric ganglion of adult lobsters^16^, and here we confirmed its abundance in the eye of *P. ornatus* phyllosoma. Potassium appears to have increasing abundance in the eye as phyllosoma develop with increasing concentrations from stage 3 to stage 8, and decreasing abundance in the mouth with decreasing concentrations from stage 3 to stage 8. The increasing potassium concentrations in the eyes are possibly related to the increasing reliance on vision detection for prey capture as the phyllosoma develop. The decrease in concentrations of potassium in the mouth could be due to the increase in size of the mouth parts and the increase in abundance of other elements (such as calcium).

The highest concentrations of calcium were located in areas where greater amounts of calcified exoskeleton would be expected, namely the mouth and the tail. A clear calcium exoskeleton outline of the organism was observed around the limbs and body of phyllosoma. Calcium outlined the maxilla with bidentate appendages in the mouth in stage 3, which changed to a maxilla with tridentate appendages in stage 4 and again in stage 8. The marked increased calcium hardening of the mouthparts in stage 8 is consistent with the dietary shift toward larger and coarser prey reported for later stages of phyllosoma.^48^ The hardness of calcium-based material is likely to be important for these parts of the mouth that must be resistant to friction and wear as they collect and process food. This study emphasizes the important structural role of calcium for phyllosoma larvae.

As has been reported previously for the setae of other crustaceans, bromine accumulated in the setae of *P. ornatus* phyllosoma, suggesting that it is used in the noncalcified exoskeleton.^34^ The bromine containing noncalcified exoskeleton must allow significant bending and stretching to be suitable for fast movement in water, without cracking, breaking or becoming damaged. The stems of the limbs are structurally much harder with a calcified exoskeleton, but the hair-like filaments extending from these limbs are invisible in the calcium map, with bromine clearly visible instead (Fig. 7). Neatly arranged setae for imaging were uncommon due to the sample preparation method, but fortunately images of setae were still able to be obtained from all three stages of phyllosoma development. As has been reported previously in the jaws of the polychaete *Nereis*, bromine concentrations were highest at the center and base of the mouth.^33^ Again this is likely to be because brominated, noncalcified fibers composed of biopolymers such as collagen and chitin, that are more flexible and capable of rapid movement are needed at the base, while hardened calcified material is required at the tips for resistance to friction and wearing.

Within the eye and mouth of *P. ornatus* phyllosoma, tissues containing high amounts of bromine were observed. This may be because bromine is also involved in the crosslinking of collagen in phyllosoma. Collagen is essential for connective tissues and muscle function in animals and it has been demonstrated that bromine is essential for the production of collagen in drosophila, which is essential for their survival and healthy development.^31^ Future studies could attempt to measure collagen concentrations in phyllosoma and investigate how this correlates with bromine concentrations, or investigate the effect of bromine depletion on collagen production, development and survival for phyllosoma.

Three trace elements were unmistakably observed in the *P. ornatus* phyllosoma, namely copper, zinc, and arsenic. Copper distributions emphasized the location of the hepatopancreas of the larvae. The branched structure within the hepatopancreas became more apparent as the larvae developed from stage 3 to stage 4 and then again to stage 8. As mentioned earlier, essential copper (which has many important functions) for lobsters must be obtained through the diet, as there are insufficient quantities of it to be obtained from seawater. Therefore, phyllosoma have to be very effective at extracting copper from copper-rich food, such as other planktonic invertebrates with hemocyanin. This may explain the significantly higher concentrations of copper present in the mouth and the hepatopancreas compared to other areas of the organism and why the concentrations of copper in the hepatopancreas significantly increased as the phyllosoma developed from stage 4 to stage 8 in particular, as they increased the quantity of food they consumed. Furthermore, as the phyllosoma grow from stage 3 to stage 8, there is a considerable increase in the overall blood volume, and therefore an increase in the copper concentrations could be expected as the concentration of hemocyanin increases. Future studies could assess the concentrations of hemocyanin protein in phyllosoma using standard biological methods and further investigate the correlation between copper and hemocyanin.

There were no significant differences in zinc concentrations as phyllosoma progressed from stage 3 through to stage 8 for any of the locations examined. However, at all stages, zinc accumulated significantly more in the eyes and mouth of phyllosoma than in the hepatopancreas and tail. There is a strong correlation between the distribution of zinc and bromine throughout all phyllosoma examined. This has been reported previously, where zinc plays a role in the noncalcified bromine containing crosslinked protein casing of invertebrates. Birkedal *et al.*^49^ found that the jaws of the marine polychaete *Nereis* are hard without mineralization. They found that bromine was associated with a variety of post-translationally modified amino acids, namely dibromohistidine, bromoiodohistidine, bromoiodotyrosine, chlorobromotrityrosine, and bromoiodotrityrosine, which had not previously been reported.^49^ Zinc, chloride, and concentrations of these post-translationally modified amino acids were key determinants of jaw hardness and important for protein crosslinking.

Arsenic accumulated significantly more in the eyes of phyllosoma than in other areas, but it remains unknown whether this arsenic was problematic or not. There was also a potential correlation between the distributions of zinc and arsenic in some of the samples, particularly in the eyes and mouth of stage 8 phyllosoma, but it remains unclear why this was the case. As mentioned earlier, arsenobetaine is the predominant arsenic compound found in marine animals, which has been shown to have a relatively low toxicity to aquatic organisms, and is likely to be the form of arsenic present in these phyllosoma.^30^ Further investigations into the chemical state of the arsenic could potentially be attempted using high performance liquid chromatography (HPLC)-MS or X-ray absorption near edge structure (XANES) on the XFM beamline, but due to the anticipated large variety and low concentrations of potential arsenic compounds that could be present, distinguishing and quantifying the proportions of the different arsenic compounds would be very difficult to achieve, particularly in such small samples.

Some of the variation in the measured concentrations could have arisen from the assumption that the specimens are uniformly 40 µm thick. It is important to note that in thinner areas of the samples the elemental concentrations may be underestimated and thicker areas overestimated because of this. Further investigations are necessary in order to determine whether the cause of differences in concentrations were solely due to physical differences in concentrations in the samples, or differences in thicknesses or compositions and resulting differences in measurements of those concentrations. Future studies could analyse bulk quantities of phyllosoma, ascertain bulk concentrations using ICP–MS, and compare bulk concentrations to those observed in individual phyllosomata using XFM and GeoPIXE software fitting.

## Conclusion

In summary, synchrotron XFM was conducted for the first time on phyllosoma, the larval stage of *P. ornatus*, which is a commercially important spiny lobster species. In this study, the elemental distributions present in *P. ornatus* phyllosoma were examined as they developed from stage 3 to stage 4 and then to stage 8. Elements accumulated in certain locations within phyllosoma, providing insight into their likely biological role for these organisms, about which very little is currently known. This information may be useful for the application of dietary supplementation in the future to closed larval cycle lobster aquaculture operations.

## Supplementary Material

mfad038_Supplemental_File

## Data Availability

The data underlying this article are available in the article and in its online supplementary material.
