# Supplementary material for: Examining the inorganic elemental composition of lobster phyllosoma (Panulirus ornatus) with X-ray fluorescence microscopy
Source: Metallomics. 2023 Jun 16;15(6):mfad038. doi: 10.1093/mtomcs/mfad038 (PMC10311473; doi:10.1093/mtomcs/mfad038)
Supplement: mfad038_Supplemental_File [file mfad038_supplemental_file.docx]

# Supplementary information

## Rearing water metal concentrations

ICPMS results confirmed that all seawater samples collected had similar concentrations of the elements studied with XFM. There were no significant differences in the concentrations of elements in the seawater collected from tanks that contained different stages of phyllosoma (P > 0.05).

## Thickness Estimation

Thickness estimation was performed using the measured x-ray transmission T at 18500 eV. The equation describing the exponential decrease in intensity with distance travelled is I(t) = Ioe-µt, where Io is the incident intensity, µ is the linear attenuation coefficient, and t is the sample thickness. With rearrangement of the equation, and substituting transmission T = I/Io, t can be calculated by t = -ln(T) / µ.

Due to the high transmission through the lobster samples, we performed an initial measurement to test to the suitability of the method with a well-specified material. The transmission at 18500 eV through Kapton (polyimide) adhesive tape was used to calculate its thickness. The Kapton tape consisted of two layers: a 30 µm thick Kapton film (C_22_H_10_N_2_O­, density = 1.43 g cm^-3^) and 45 µm thick silicone adhesive layer (SiOC2H6, density = 0.96 g cm-3). The NIST X-Ray Form Factor, Attenuation, and Scattering Tables (FFAST) database [1-3] was used to determine the linear attenuation coefficient of the Kapton tape, based on the compositions and densities of the two layers. The linear attenuation coefficient value of µ = 1.06 cm-1 was calculated at 18500 eV. The measured transmission through the 75 µm thick Kapton tape was T = 0.9910, corresponding to a calculated thickness of 0.00853 cm or 85 µm. We considered this to be satisfactory to proceed with the transmission through the lobsters as a means of determining their thickness.

The assumed composition and density of the lobsters was C_25_H_40_O_7_Ca and 1.42 g cm^-3^. Using the same method as for the Kapton tape above, a linear attenuation coefficient of 2.57 cm^-1^ was calculated with the NIST X-Ray Form Factor, Attenuation, and Scattering Tables (FFAST) database at 18500 eV.

The transmission was averaged over several areas of the lobsters and are shown in Table S1. The projected thicknesses at first seem less than expected. For example, the eye stems for the larger lobsters have a diameter of 200 µm but have a calculated thickness of 53 µm through the centre. We attribute this to the freeze-drying process creating a more porous structure [4]. Based on these findings, a thickness of 40 µm was used in the analysis.

Table S1. Lobster projected thickness calculation based on X-ray transmission data at 18500 eV using a linear attenuation coefficient of 2.57 cm^-1^.

| *Body part* | *Transmission (T)* | *Projected thickness (µm)* |
| --- | --- | --- |
| *Main body segment* | 0.9968 | 13 |
| *Tail body segment* | 0.9913 | 34 |
| *Small lobster body* | 0.9925 | 29 |
| *Large lobster eye* | 0.9628 | 147 |
| *Eye stem* | 0.9866 | 53 |

## Semi-quantitative concentrations

Mean concentrations based on semi-quantitative data obtained using XFM and GEOPIXE software spectrum fitting are shown in Supplementary Figure 1.


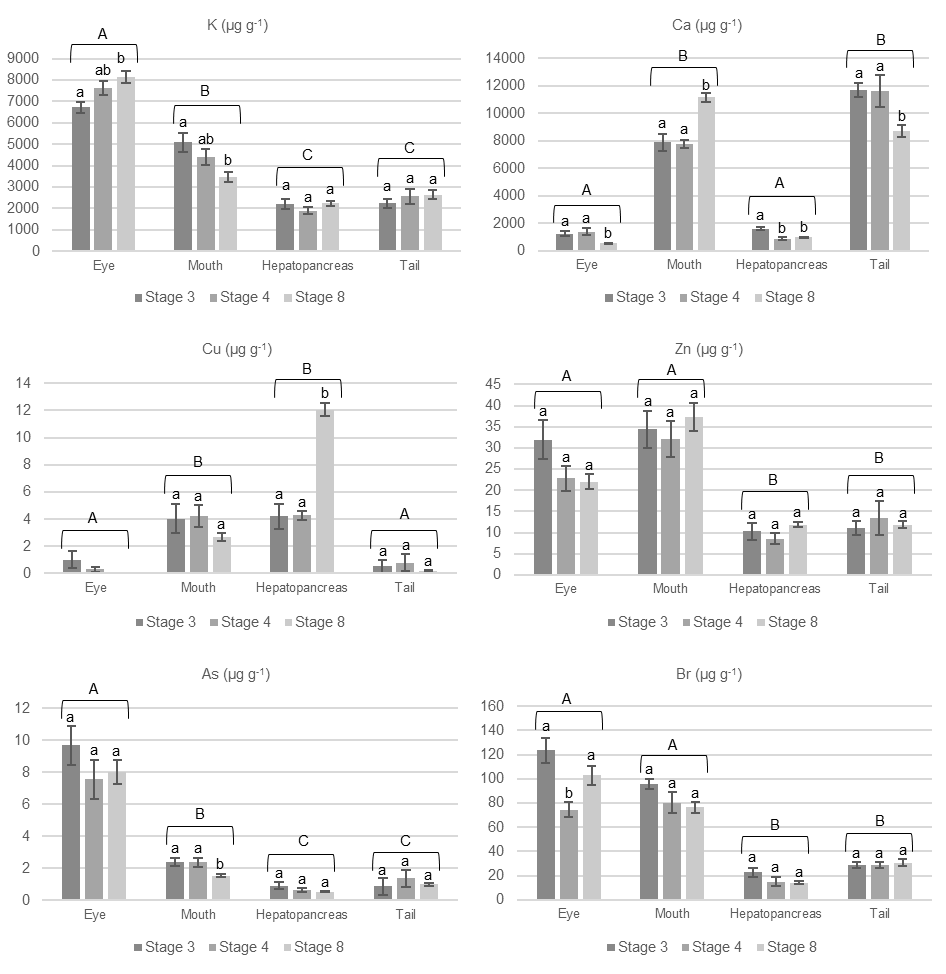


Supplementary Figure 1. Mean concentrations ± S.E. of potassium (K), calcium (Ca), copper (Cu), zinc (Zn), arsenic (As) and bromine (Br) in the eye, mouth, hepatopancreas and tail of *P. ornatus* phyllosoma larvae at stages 3, 4 and 8 of development. All concentrations are semi-quantitative and are reported in µg g^-1^. For stage 3, n = 6, for stage 4, n = 7 and for stage 8, n= 9. Different lowercase letters above bars indicate significant differences between developmental stages (Tukey’s or Dunn’s test, P < 0.05). Different uppercase letters above brackets indicate significant differences between anatomical regions (Tukey’s test, P < 0.05).

## Statistical analysis results

### Changes in concentrations with development

An analysis of variance on the elemental concentrations obtained from XFM yielded significant variation among the three examined developmental stages of phyllosoma. There were significant differences in the mean concentrations of potassium, calcium, copper, arsenic and bromine among the three developmental stages (P < 0.05). There were no significant differences in the mean zinc concentrations among the developmental stages examined (P>0.05).

A post-hoc Tukey test showed that as phyllosoma progressed from stage 3 to stage 8, the mean concentration of potassium significantly increased in the eye (q = 4.54, P < 0.05) but significantly decreased in the mouth (q = 4.655, P < 0.05). There were no significant differences in the mean potassium concentrations in the hepatopancreas or the tail among the examined stages of development (P > 0.05).

A post-hoc Dunn’s test (Q) and Tukey test (q) showed that as phyllosoma progressed from stage 4 to stage 8, the mean concentration of calcium significantly decreased in the eye (Q = 3.129, P < 0.05) and the tail (Q = 2.452, P < 0.05), but significantly increased in the mouth (q = 8.832, P < 0.05). As phyllosoma progressed from stage 3 to stage 4, the mean concentration of calcium significantly decreased in the hepatopancreas (q = 8.442, P < 0.05).

A post-hoc Dunn’s test showed that as phyllosoma progressed from stage 4 to stage 8, the mean concentration of copper significantly increased in the hepatopancreas (Q = 3.143, P < 0.05). There were no significant differences in the mean copper concentrations among all examined stages of development for the eye, mouth, or tail (P > 0.05).

A post-hoc Tukey test showed that as phyllosoma progressed from stage 4 to stage 8, the mean concentration of arsenic significantly decreased in the mouth (q = 4.045, P < 0.05). There were no significant differences in the mean arsenic concentrations among all examined stages of development for the eye, hepatopancreas, or tail (P > 0.05).

A post-hoc Tukey test showed that as phyllosoma progressed from stage 3 to stage 4, the mean concentration of bromine significantly decreased in the eye (q = 5.611, P < 0.05), then from stage 4 to stage 8 significantly increased (q = 3.616, P < 0.05). There were no significant differences in the mean bromine concentrations among all examined stages of development for the mouth, hepatopancreas or tail (P > 0.05).

### Differences in concentrations among anatomical regions

An analysis of variance on the concentrations obtained from XFM yielded significant variation among different anatomical regions of phyllosoma. There were significant differences in the mean concentrations of potassium, calcium, copper, zinc, arsenic and bromine among the eyes, mouths, hepatopancreases and tails of all phyllosoma examined, regardless of developmental stage (P < 0.05).

A post-hoc Tukey test showed that mean potassium concentrations were significantly higher in the eyes of phyllosoma than in all other regions examined (q = 4.385, P < 0.05). However, the mean potassium concentrations were significantly higher in the mouth than the hepatopancreas and tail (q = 6.213, P < 0.05 and q = 4.665, P < 0.05, respectively). There was no significant difference in the mean potassium concentration between the hepatopancreas and the tail (q = 1.548, P > 0.05).

A post-hoc Tukey test showed that mean calcium concentrations were significantly higher in the mouth and tail of phyllosoma than in the eye and hepatopancreas (P < 0.05). There were no significant differences in the mean calcium concentrations between the eye and the hepatopancreas (q = 1.260, P > 0.05) or between the mouth and the tail (q = 1.202, P > 0.05).

A post-hoc Tukey test showed that mean copper concentrations were significantly higher in the mouth and hepatopancreas of phyllosoma than in the eye and tail (P < 0.05). There were no significant differences in the mean copper concentrations between the mouth and the hepatopancreas (q= 2.896, P >0.05) or between the eye and the tail (q = 0.709, P > 0.05).

A post-hoc Tukey test showed that mean zinc concentrations were significantly higher in the eye and mouth of phyllosoma than in the hepatopancreas and tail (P < 0.05). There were no significant differences in the mean zinc concentrations between the eye and the mouth (q = 2.575, P > 0.05) or between the hepatopancreas and the tail (q = 0.764, P > 0.05).

A post-hoc Tukey test showed that mean arsenic concentrations were significantly higher in the eye of phyllosoma than in all other regions examined (q = 4.506, P < 0.05). However, the mean arsenic concentrations were significantly higher in the mouth than in the hepatopancreas and tail (q = 6.401, P < 0.05 and q = 4.198, P < 0.05, respectively). There was no significant difference in the mean arsenic concentration between the hepatopancreas and tail (q = 2.203, P > 0.05).

A post-hoc Tukey test showed that the mean bromine concentrations were significantly higher in the eye and mouth of phyllosoma than in the hepatopancreas and tail (P < 0.05). There were no significant differences in the mean bromine concentrations between the eye and mouth (q = 1.427, P > 0.05) or between the hepatopancreas and tail (q = 3.038, P > 0.05).

# Data availability

The data underlying this article are available in the article and in its online supplementary material.

# References

1. Chantler, C., K. Olsen, R. Dragoset, J. Chang, A. Kishore, S. Kotochigova, D. Zucker, and X.-r.F. Factor, Attenuation and Scattering Tables*.* *Detailed tabulation of atomic form factors, photoelectric absorption and scattering cross section, and mass attenuation coefficients for*, 2005(1-92): p. 1-10.

2. Chantler, C.T., Theoretical form factor, attenuation, and scattering tabulation for Z= 1–92 from E= 1–10 eV to E= 0.4–1.0 MeV*.* *Journal of Physical and Chemical Reference Data*, 1995. **24**(1): p. 71-643.

3. Chantler, C.T., Detailed tabulation of atomic form factors, photoelectric absorption and scattering cross section, and mass attenuation coefficients in the vicinity of absorption edges in the soft X-ray (Z= 30–36, Z= 60–89, E= 0.1 keV–10 keV), addressing convergence issues of earlier work*.* *Journal of Physical and Chemical Reference Data*, 2000. **29**(4): p. 597-1056.

4. Krokida, M., V. Karathanos, and Z. Maroulis, Effect of freeze-drying conditions on shrinkage and porosity of dehydrated agricultural products*.* *Journal of Food Engineering*, 1998. **35**(4): p. 369-380.
